# Validity of the peak velocity to detect physical training improvements in athymic mice

**DOI:** 10.3389/fphys.2022.943498

**Published:** 2022-08-24

**Authors:** Maurício Beitia Kraemer, Karen Christine Silva, Camila Cunha França Kraemer, Juliana Silva Pereira, Ivan Gustavo Masseli dos Reis, Denise Gonçalves Priolli, Leonardo Henrique Dalcheco Messias

**Affiliations:** ^1^ Research Group on Technology Applied to Exercise Physiology (GTAFE), Laboratory of Multidisciplinary Research, São Francisco University, Bragança Paulista, Brazil; ^2^ Coloproctology Service of the Federal University of São Paulo, São Paulo and Faculty of Health Sciences Pitágoras de Codó, São Paulo, Brazil

**Keywords:** neoplasm, athymic mice, aerobic power, training load, exercise

## Abstract

This study comprises two complementary experiments with athymic Balb/c (Nu/Nu) mice. In experiment 1, the aim was to verify the reproducibility of the peak velocity (V_Peak_) determined from the incremental test. The second experiment aimed to assess the V_Peak_ sensitivity to prescribe and detect modulations of the physical training in athymic nude mice. Sixteen mice were submitted to two incremental treadmill tests separated by 48-h (Experiment 1). The test consisted of an initial warm-up of 5 minutes. Subsequently, animals initiated the tests at 8 m min^−1^ with increments of 2 m min^−1^ every 3 minutes. The V_Peak_ was determined as the highest velocity attained during the protocol. In experiment 2, these animals were randomly allocated to an exercise group (EG) or a control group (CG). The training protocol consisted of 30-min of treadmill running at 70% of the V_Peak_ five times a week for 4 weeks. High indexes of reproducibility were obtained for V_Peak_ (Test = 19.7 ± 3.6 m min^−1^; Retest = 19.2 ± 3.4 m min^−1^; *p* = 0.171; effect size = 0.142; r = 0.90). Animals from the EG had a significant increase of V_Peak_ (Before = 18.4 ± 2.7 m min^−1^; After = 24.2 ± 6.0 m min^−1^; *p* = 0.023). Conversely, a significant decrease was observed for the CG (Before = 21.1 ± 3.9 m min^−1^; After = 15.9 ± 2.7 m min^−1^; *p* = 0.038). The V_Peak_ is a valid parameter for exercise prescription in studies involving athymic nude mice.

## Introduction

The “hairless” Balb/c (*Mus musculus*) was initially described in 1962 by Dr. Grist of the Virus Laboratory at Ruchill Hospital, Glasgow (Issacson and Cattanach, 1962). At first inspection, the absence of a hair coat was the most evident difference in this mouse. Further, such characteristics and their pleiotropic impacts on fertility, mortality, histology, morphology, and genetics were explored by Flanagan, (1966). This author theorized that these deformities were produced by a recessive gene known as “nude” and symbolized as *nu* (thus the designation BALB/c (Nu/Nu) mice). Along with the hairlessness, these animals showed slow growth, poor fertility, and the development of liver disease, which ultimately led to premature death before 25–30 weeks. Two years later, a reinvestigation revealed that BALB/c (Nu/Nu) mice lack the thymus (Pantelouris, 1968), being unable to produce T-cells and then characterized as immunodeficient or athymic.

This immunodeficient feature made the athymic mice relevant to the study of drug action (Juul et al., 1992) and development (Kelland, 2004), besides cytotoxicity research (Budzynski and Radzikowski, 1994) and several diseases, including autoimmune (Volpe et al., 1993) and cancer (Szadvari et al., 2016). Regarding the latter, this model has been largely applied in distinct contexts associated with neoplasms, including breast (Osborne et al., 1985), colorectal (Giavazzi et al., 1986; da Silva et al., 2022), prostate (van Weerden and Romijn, 2000), liver (Yoysungnoen et al., 2008), lung (Liu et al., 2012), pancreatic (Zhao et al., 2013) and neurological cancers (de Oliveira et al., 2019). Therefore, based on the exponential increase in global oncology cost over the last 10 years (2011 = US$ 56 billion; 2021 = US$ 187 billion) (Statista, 2022) and the high cancer mortality rate (Moss et al., 2020), the BALB/c (Nu/Nu) mice have become an important model onto new therapeutics. Apart from the pharmacological approaches, physical exercise has been suggested as a relevant component of cancer treatment (Idorn and Thor Straten, 2017; Ferioli et al., 2018; Hojman et al., 2018; Esteves et al., 2021). However, the scientific community lacks information about the impact of manipulating training variables on cancer in humans (Kraemer et al., 2022), and the same is true for experiments with Balb/c (Nu/Nu). Although some reports have submitted these rodents to physical exercise (Jones et al., 2005; Lee et al., 2017; Lee et al., 2018; Hagar et al., 2019), a variety of non-individualized protocols were conducted, hampering further conclusions on the influence of training intensity and volume for these animals.

Several protocols have been carried out with rodents to identify physiological parameters for individual control and prescription of physical training. The maximal lactate steady state (MLSS) and lactate minimum test (LMT) may be valid approaches for this purpose (Gobatto et al., 2009; de Araujo et al., 2012; Messias et al., 2017; Rodrigues et al., 2017). On the other hand, given the average total blood volume of a mouse (72–80 ml/kg) (Mitruka and Rawnsley, 1977; Diehl et al., 2001) and the potential immunological window resulting from the invasive procedures, the need for multiple blood collections may stymie these protocols for Balb/c (Nu/Nu). Moreover, Lonbro et al. (2019) verified large variations in blood lactate kinetics of athymic mice submitted to exercise, hampering the MLSS determination. According to these authors, the inter-strain variability along with handling and pacing these mice during exercise may influence the blood lactate levels, possibly explaining the ineffective MLSS determination. In this sense, non-invasive tests such as the critical velocity concept (CV) can alternatively provide reliable parameters for training controlling, and prescription of rodents (Billat et al., 2005; Manchado-Gobatto et al., 2010). However, a minimum of three predictive trials may preclude this test in studies with tumor growth, which have a limited period for cancer implantation.

Due to the model’s idiosyncrasies, alternative ways to determine training parameters for BALB/c (Nu/Nu) mice are required. In this way, the peak velocity (VPeak) can be measured in just one incremental test without invasive procedures. This parameter is largely used for humans (Bassett and Howley, 2000; Bosquet et al., 2002), but Picoli et al. (2018) recently demonstrated that V_Peak_ is as sensitive as the maximal oxygen consumption (VO_2max_) to prescribe and monitor exercise training in Swiss male mice. Nevertheless, no study has verified if this parameter is reproducible and appropriate to prescribe the exercise intensity for the immunodeficient BALB/c (Nu/Nu) mice. For the first time, we provide two complementary experiments that span this gap. The findings of this study provide new avenues for studying and improving physical training using the BALB/c (Nu/Nu) mice. Overall, this study aims to verify the VPeak reproducibility (Experiment 1) and sensitivity to prescribe and detect modulations of the physical training (Experiment 2) in BALB/c (Nu/Nu) mice. Despite the athymic characteristic, we hypothesize that this parameter based on non-invasive and 1-day protocol is reproducible and appropriate to prescribe exercise intensity for these animals.

## Material and methods

### Animals

Sixteen 1-month BALB/c (Nu/Nu) mice (8 male and 8 female) from Charles River were used in this study. Throughout the experiments the rodents were kept in a room with a controlled environment, including temperature (23 ± 1°C), relative humidity (45–55%), noise (<80 dB), and a 12:00 h light/dark cycle (illumination from 6.00 a.m. to 6.00 p.m.). Mice were housed one per polyethylene cage with 40-cm length, 33-cm width, and 16-cm height. Food (Neovia, São Paulo, Brazil), and water were provided *ad libitum* and autoclaved before use. Cages were replaced each week and were also autoclaved.

### Experimental design

**FIGURE 1:**
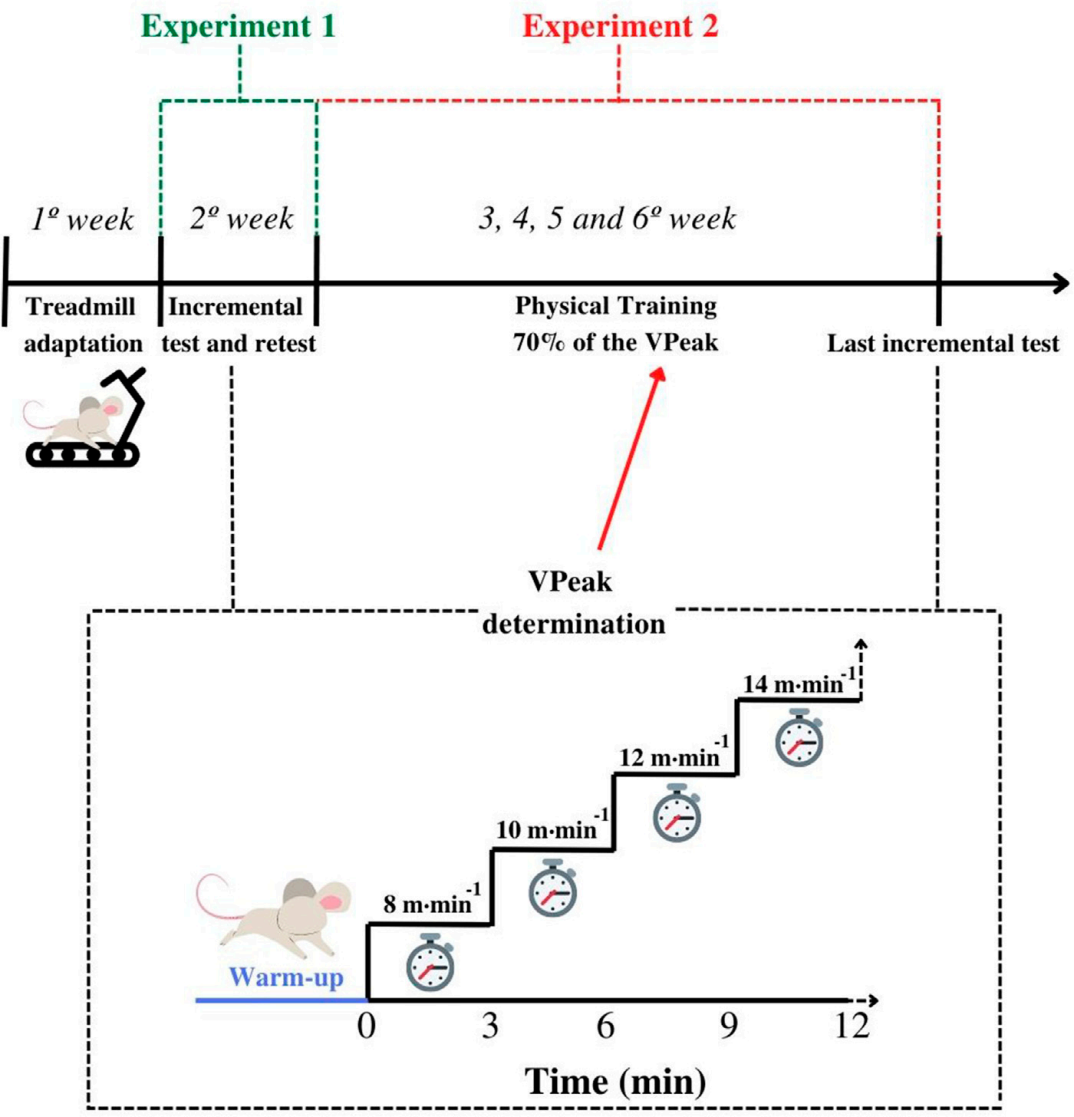
Experimental design of the study. After adaptation to the treadmill, mice were submitted to the incremental test and retest for the peak velocity (V_Peak_) determination (Experiment 1—reproducibility of the incremental test results). Animals from the exercise group were submitted to 4 weeks of physical training based on 70% of the V_Peak_. During the same period, the controls did not perform any physical exercise. After the sixth week, both controls and exercised were submitted to the last incremental test (Experiment 2—sensitivity of the incremental test parameters). Note that controls were again submitted to a new treadmill adaptation the week before the last incremental test.

Animals were randomly allocated into the exercise group (EG) (*n* = 8) or control group (CG) (*n* = 8), regardless of sex. Before the beginning of the intervention, both groups were adapted to the treadmill exercise for 5 days. On the first day, animals were submitted to 10-min continuous running exercises at 6 m min^−1^. Five minutes and 1 m min^−1^ were added per day, until the last adaptation session of 30 min at 10 m min^−1^. In the next week, the athymic rodents were submitted to two incremental tests (test and retest) separated by 48 h (Experiment 1). During the subsequent 4 weeks, the EG performed individualized training, while animals in the CG remained without physical effort (Experiment 2) (Figure 1). Three days after the intervention period, animals from both groups were submitted to the last incremental test and then euthanized. Throughout the experiment, body mass, food, and water intake were individually measured twice a week (i.e., Monday and Friday), at the same time (11:00 a.m.). The delta between Monday and Friday indicated the body mass variation inside the week as well as the intake of food and water. The delta between Friday and the next Monday indicated the body mass, food, and water intake variations during the weekends. Moreover, the food and water intake were normalized by the body mass using the following equation:

$$\mathrm{Normalized}\;\mathrm{intake}=\left(\mathrm{IW-FW}\right)\mathrm{xN}D^{\mathrm{-1}}\mathrm{xB}M^{\mathrm{-1}}$$

Where IW refers to the initial weight of food or water, FW is equal to the final weight of food and water, ND is the number of days, and BW is the body mass.

### Experiment 1—Reproducibility of the incremental test parameters

In the week before the intervention period, the animals were submitted to two similar treadmill incremental tests separated by 48 h. Considering that at this moment every animal was in the same condition (i.e., with or without the training intervention), we clustered all rodents (*n* = 16) to verify the reproducibility of the incremental test parameters. Tests were performed at the same time (2:00 p.m.) and by the same researcher in blind condition. The incremental test consisted of an initial warm-up of 5 minutes (3 minutes at 6 m min^−1^ and the remaining 2 minutes at 7 m min^−1^). Subsequently, animals initiated the tests at 8 m min^−1^ with increments of 2 m min^−1^ every 3 minutes. The exhaustion criteria consisted of the incapacity to keep running for more than 10-s (Kregel et al., 2006).

The total time and the peak velocity (V_Peak_) were retrieved for testing the reproducibility of the incremental test. Total time was defined as the amount of time—except for the warm-up—those animals performed during the test. V_Peak_ was calculated by the equation of Kuipers et al. (2003):

$$V_{\mathrm{peak}}=\mathrm{V+t/Tx}\;\mathrm{velocity}\;\mathrm{increment}$$

Where V is the velocity of the last completed stage (m min^−1^), t is the time (s) of the uncompleted stage, and T is the total time of the stage (180-s).

### Experiment 2—Sensitivity of the incremental test parameters to the individualized training

BALB/c (Nu/Nu) mice of the EG were submitted to 4 weeks of individualized training on a treadmill. The training sessions occurred five times a week (Monday to Friday) with a volume of 30-min per day. The intensity was established at 70% of the V_Peak_ determined in the first incremental test (Picoli et al., 2018). Based on the reproducibility results—which will be detailed in a further section—only two Balb/c (Nu/Nu) presented a considerable reduction in the second V_Peak_ (i.e., retest). Suggestively, these animals could not be fully recovered from the first incremental test. Therefore, and to avoid this effect, the V_Peak_ from “test” was chosen rather than those from “retest”. After the training period, animals from both groups were again submitted to the incremental test.

The ratio between the performed to the predicted volume was individually quantified. The daily training load was obtained by the product between training volume (min) and intensity (70% of the V_Peak_) and expressed in arbitrary units (A.U). Based on the daily load, the mean weekly load was calculated for each week. Training monotony (i.e., loading fluctuations) was calculated as the mean weekly load/standard deviation of the total weekly load. Strain (i.e., overall stress generated by training) was calculated as total weekly load/monotony.

### Statistical analyses

The statistical analyses were conducted using a statistical software package (STATISTICA 7.0, Statsoft, OK, United States). Levene and Shapiro-Wilk tests confirmed the homogeneity and normality of our data. Mean and standard deviation (SD) were used for all variables. Coefficient of variation (CV) was calculated based on the mean and SD. Reproducibility analysis was procced by *t*-test for dependent samples, effect size (ES) (Cohen, 1994), Pearson product-moment, and the Bland-Altman approach (Bland and Altman, 1986). Cohen’s categories used to evaluate the magnitude of the ES were: small if 0 ≤ |d| ≤0.5; medium if 0.5 < |d| ≤ 0.8; and large if |d| > 0.8. Comparison among the incremental test results (total time and V_Peak_) as well as body mass, food, and water intake for both EG and CG before and after the intervention period was procced by two-way ANOVA. The Newman Keuls post-hoc was considered in all analyses of variance. The ANOVA for repeated measures was adopted for the comparison of the mean weekly load calculated from the EG. The body mass comparison during the experiment was procced by three-way ANOVA. We opted to provide the raw body mass values rather than the variation through weeks and weekends to preserve the magnitude of this parameter. Therefore, moment (weeks), intervention (exercise vs. control), and the day of the week (Beginning/Monday vs. Ending/Friday) were considered factors. The area under the curve was calculated by a trapezoidal approach using the GraphPad Prism 5.0 software (GraphPad Software, CA, United States). In all cases, statistical significance was set at *p* < 0.05.

## Results

### Experiment 1—Reproducibility of the incremental test parameters

**FIGURE 2:**
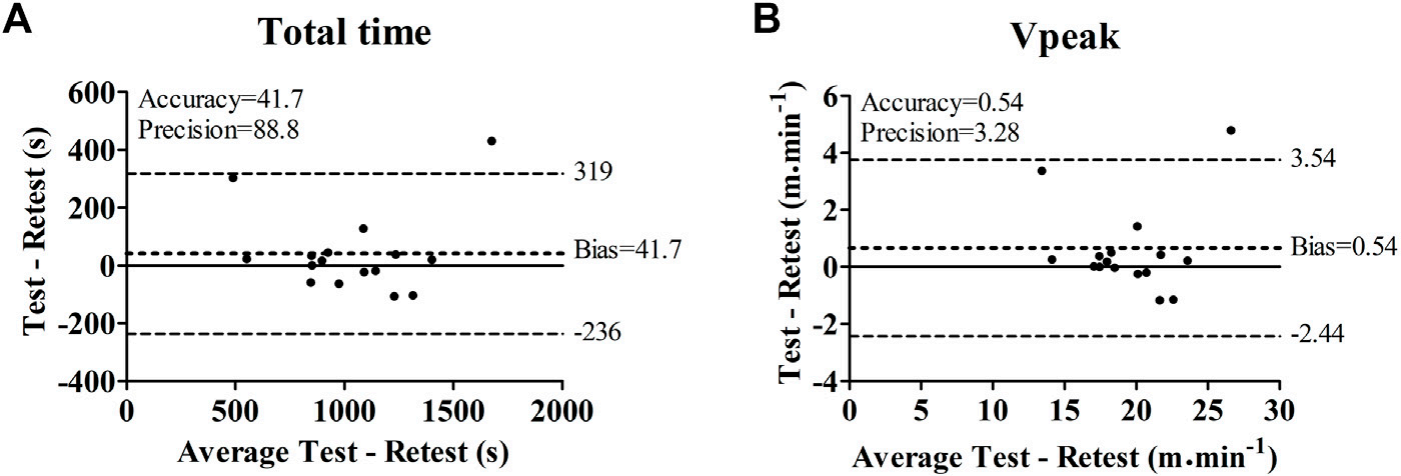
Bland-Altman comparison between the incremental test and retest in terms of total time **(A)** and peak velocity (Vpeak) **(B)**.

Table 1 present the results from the comparison between the incremental test and retest. Both total time (test—range = 563–1891 s; CV = 30.3%/retest—range = 540–1460 s; CV = 30.1%) and V_Peak_ (test—range = 15.1–29.1 m min^−1^; CV = 18.0%/retest—range = 14.0–24.2 m min^−1^; CV = 17.8%) were similar, presented a small ES and were significantly correlated between evaluations. Bland-Altman analysis showed good accuracy and precision for both total time and V_Peak_ obtained in the incremental tests (Figure 2). Only two BALB/c (Nu/Nu) mice (Animals 14 and 15) presented a large reduction (Animal 14—total time = −303 s; V_Peak_ = −3.3 m min^−1^; Animal 15—total time = −431 s; V_Peak_ = −4.7 m min^−1^) between the test and retest. For 86% of the sample, the bias of total time and V_Peak_ were 4 s and 0.04 m min^−1^, respectively.

**TABLE 1:** Reproducibility parameters between the incremental test and retest results.

N = 16	Test	Retest	*p*	ES	r (*p*)
Total time (s)	1055 ± 320	1013 ± 305	0.148	0.134	0.88 (<0.000)
Vpeak (m/min)	19.7 ± 3.6	19.2 ± 3.4	0.171	0.142	0.90 (<0.000)

ES, effect size; Vpeak—peak velocity determined from the incremental tests; *p* < 0.05.

### Experiment 2—Sensitivity of the incremental test parameters to the individualized training

**FIGURE 3:**
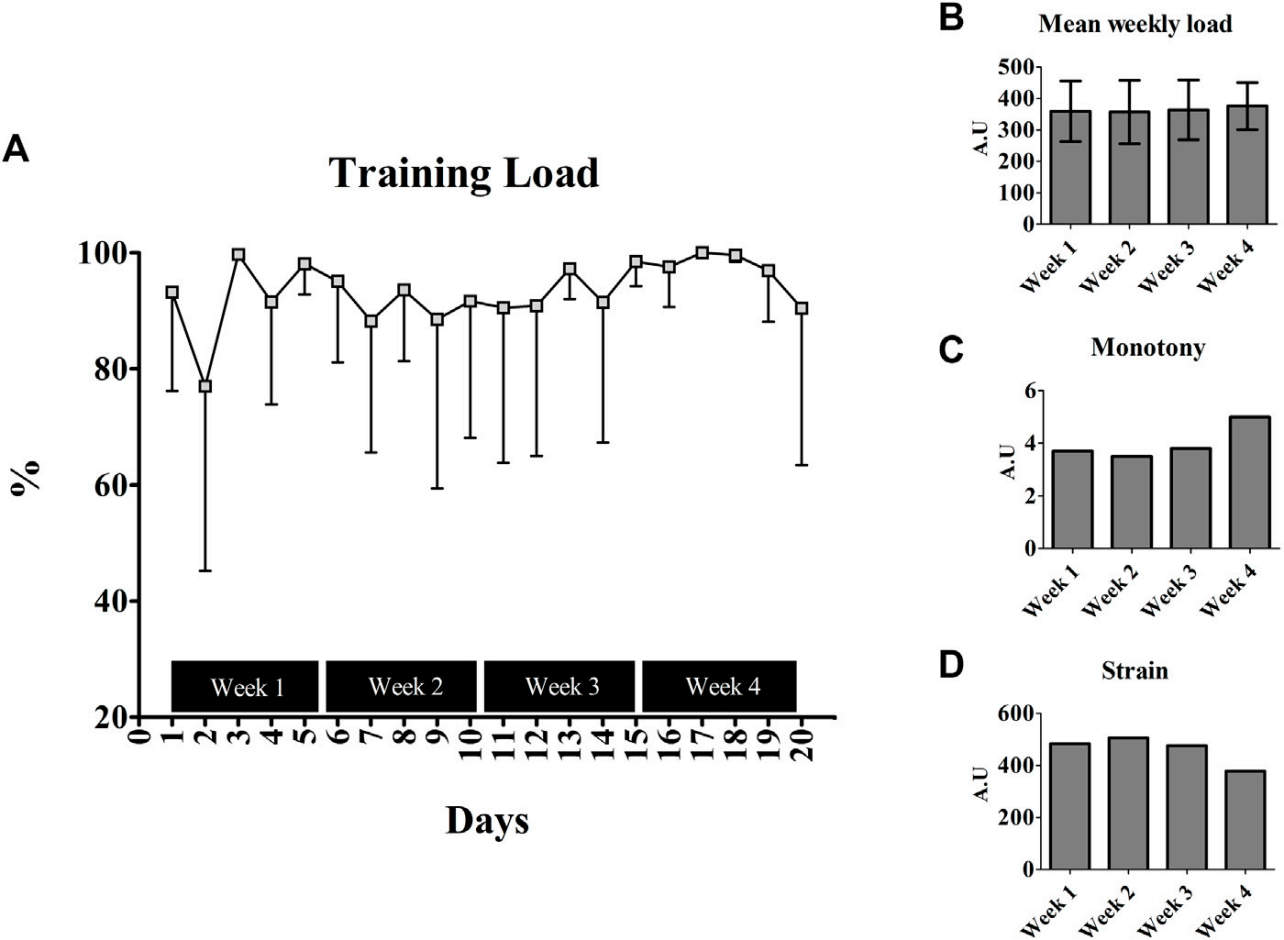
Training parameters measured from the Balb/c (Nu/Nu) submitted to physical exercise; **(A)** Training load across the training intervention; **(B)** Mean weekly load calculated from the days inside weeks; **(C)** Monotony calculated throughout the training; **(D)** Strain measured during the training intervention.

The training variables measured from the EG are presented in Figure 3. Apart from the second, seventh, and ninth days, the BALB/c (Nu/Nu) mice performed 90% of the predicted load during the intervention period (Figure 3A). No difference was observed for the weekly load throughout the training (*p* = 0.395) (Figure 3B). The monotony was unchanged during the first 3 weeks (Week 1 = 3.7; Week 2 = 3.5; Week 3 = 3.8 A.U), but an increase of ∼34% was observed in the last week (5.0 A.U) (Figure 3C). Opposite scenario was visualized for strain, with a decrease of ∼22% in last week (Week 1 = 483.58; Week 2 = 506.87; Week 3 = 477.05; Week 4 = 378.53 A.U) (Figure 3D).

**FIGURE 4:**
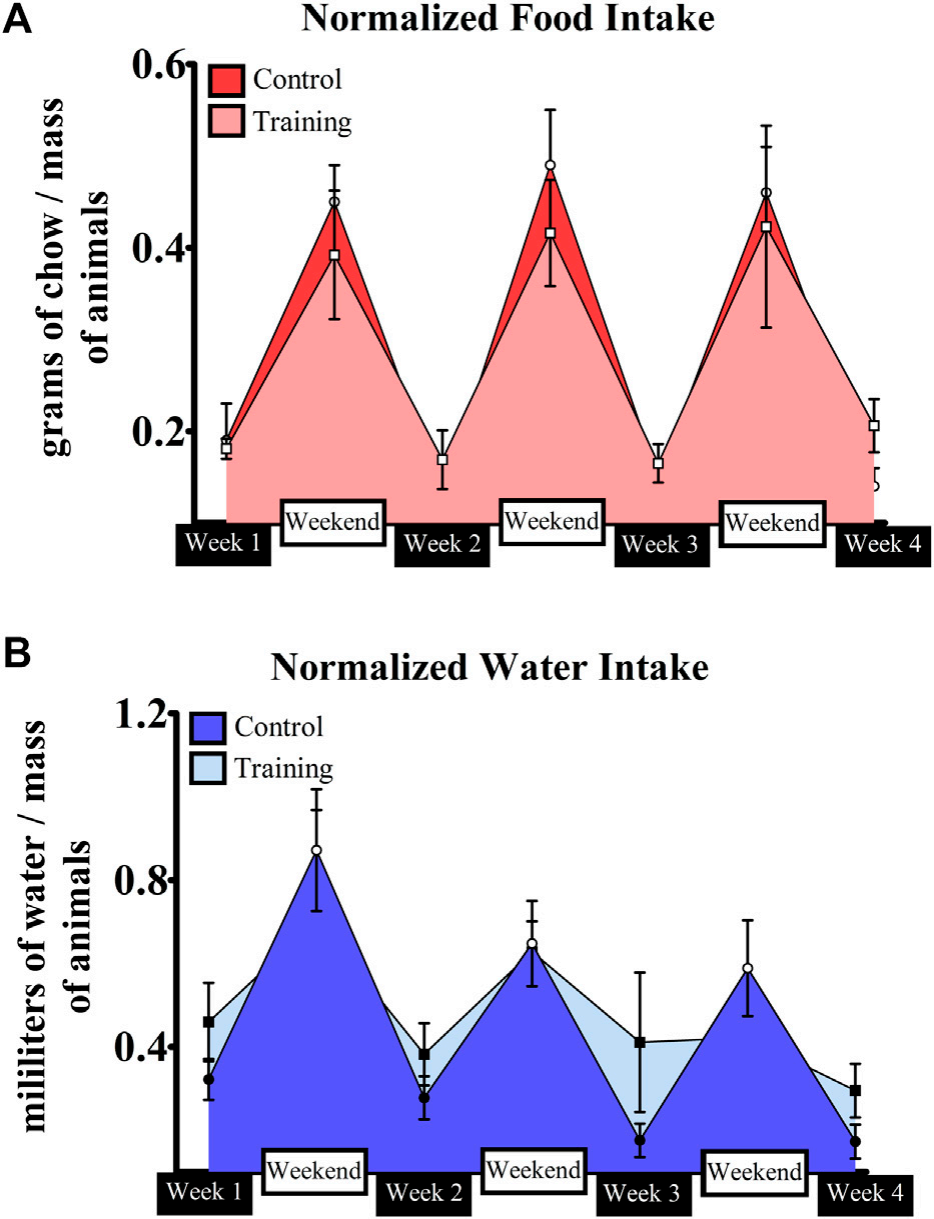
Normalized food **(A)** and water **(B)** intake by the control and intervention groups throughout the experiment.

No interaction was observed for both groups (*p* = 0.935) in terms of body mass (CG—Week 1 = 19.85 ± 2.64 g; Week 2 = 21.47 ± 2.78 g; Week 3 = 21.85 ± 2.48 g; Week 4 = 22.75 ± 2.28 g; EG—Week 1 = 23.16 ± 1.98 g; Week 2 = 23.85 ± 2.13 g; Week 3 = 23.87 ± 2.07 g; Week 4 = 24.40 ± 1.63 g). Figure 4A shows the normalized food and water intake throughout the experiment. Significant effects were observed for moment (*p* = 0.000), group (*p* = 0.002), and interaction (*p* = 0.000) in terms of food intake. Differences between weeks and weekends within the same group were visualized, but mice from the control group had higher ingestion than those trained on the weekends. Conversely, trained mice ingested more food on week 2 when compared to control (Table 2). A similar scenario was observed regarding water intake (Figure 4B). Significant effects were observed for moment (*p* = 0.000) and interaction (*p* = 0.000), but not for group (0 = 114). Animals ingested more water on weekends regardless of the group. However, a significant difference was observed between weekends 1 and 3 for trained mice. Moreover, trained mice had higher water ingestion on week 3 when compared to control (Table 3).

**TABLE 2:** Comparison between groups through the experiment in terms of food intake.

Moment	Control	Training
Week 1	0.191 ± 0.037	0.181 ± 0.011
Weekend1	0.448 ± 0.042∗	0.392 ± 0.070∗^§^
Week 2	0.131 ± 0.020	0.169 ± 0.032^§^
Weekend2	0.487 ± 0.059∗	0.416 ± 0.058∗^§^
Week 3	0.149 ± 0.017	0.165 ± 0.021
Weekend3	0.459 ± 0.051∗	0.423 ± 0.110∗^§^
Week 4	0.142 ± 0.016	0.206 ± 0.029^§^

∗Different from weeks 1, 2, 3, and 4 within the same group; ^§^Different from the respective control; *p* ≤ 0.05.

**TABLE 3:** Comparison between groups through the experiment in terms of water intake.

Moment	Control	Training
Week 1	0.322 ± 0.049	0.460 ± 0.094
Weekend1	0.872 ± 0.146∗	0.703 ± 0.265∗
Week 2	0.278 ± 0.052	0.382 ± 0.075
Weekend2	0.648 ± 0.102∗	0.628 ± 0.073∗
Week 3	0.176 ± 0.040	0.411 ± 0.167^§^
Weekend3	0.589 ± 0.115∗	0.423 ± 0.110^#^
Week 4	0.173 ± 0.041	0.295 ± 0.064

∗Different from weeks 1, 2, 3, and 4 within the same group; ^#^Different from weekend 1 within the same group; ^§^Different from the respective control; *p* ≤ 0.05.

**FIGURE 5:**
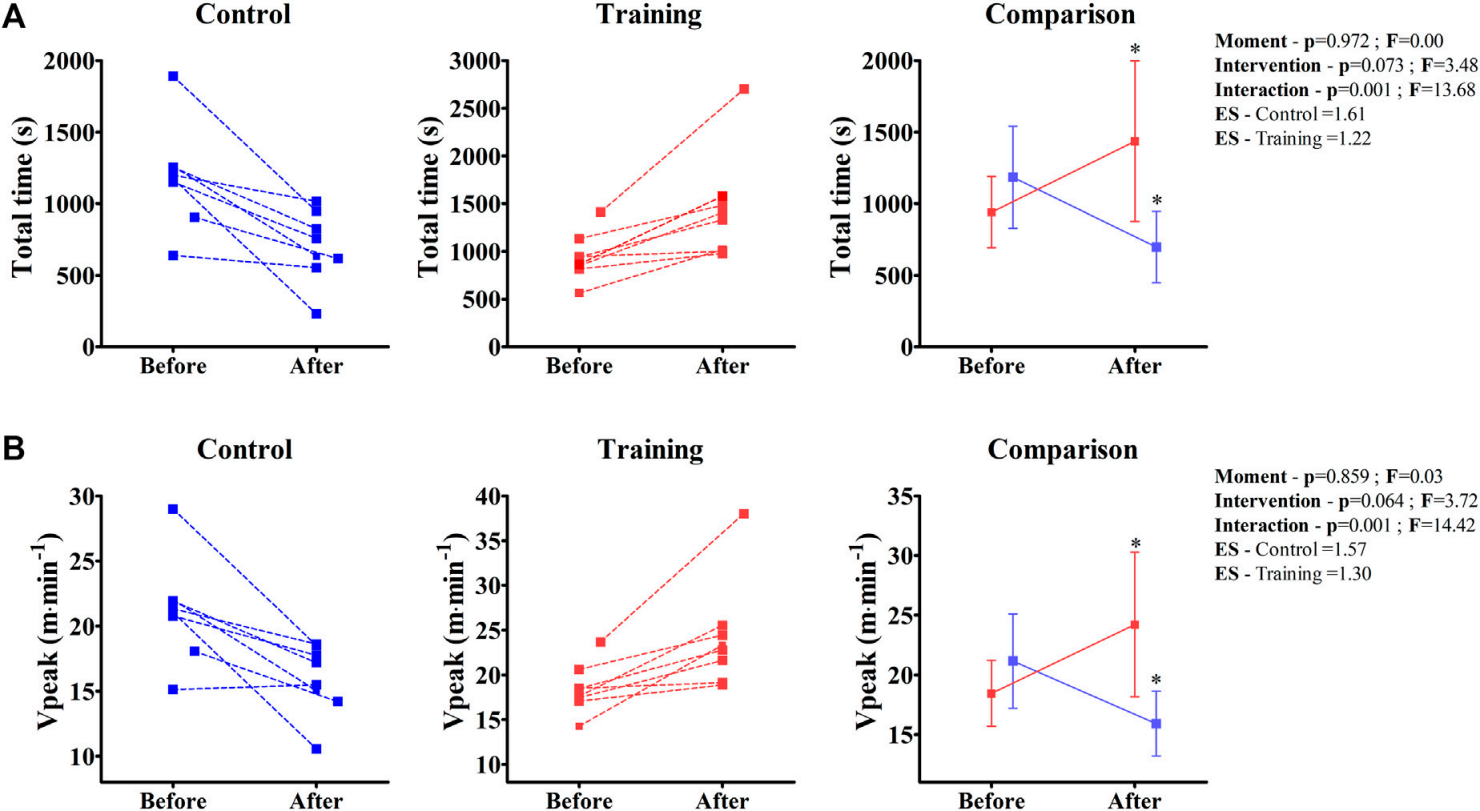
Individual values of total time **(A)** and peak velocity (Vpeak) **(B)** of control and training groups obtained from the incremental test before and after the experiment are shown in the left and middle panels. In the right panel, the ANOVA two-way comparison is shown. Note that the effect size (ES) result relates to a comparison of “Before” and “After” data within the same group; * Denotes a significant difference from “Before”; *p* < 0.05.

Both total time and V_Peak_ were significantly modified during the experiment (Figure 5). While the physical training improved these parameters in the EG group (Total time—Before = 950 ± 248 s; After = 1437 ± 561 s; *p* = 0.034/V_Peak_—Before = 18.4 ± 2.7 m min^−1^; After = 24.2 ± 6.0 m min^−1^; *p* = 0.023), the BALB/c (Nu/Nu) mice that remained without the training had a significant decrease in performance (Total time—Before = 1184 ± 355 s; After = 697 ± 248 s; *p* = 0.034/V_Peak_—Before = 21.1 ± 3.9 m min^−1^; After = 15.9 ± 2.7 m min^−1^; *p* = 0.038).

## Discussion

The hypotheses of this study were accepted. Our findings support the V_Peak_ as a method of exercise prescription and controlling for BALB/c (Nu/Nu) mice. This parameter was reproducible for 86% of the mice (14 out 16) with a very low bias between test and retest. Furthermore, the BALB/c (Nu/Nu) mice improved their VPeak by 31% after 1 month of individualized training, supporting the sensitivity feature of this parameter for this animal model.

### Experiment 1—Reproducibility of the incremental test parameters

In this study, we modified methodologic aspects to assure that all BALB/c (Nu/Nu) mice could perform the incremental protocol. Our design differs from those carried out by Picoli et al. (2018) in terms of initial velocity and intensity increment over the stages. The Swiss mice tested by these authors are frequently used in experiments involving physical exercise (Aguiar et al., 2010; De Souza et al., 2010; Wanner et al., 2014; Ludtke et al., 2020; Picoli et al., 2020; De Sousa et al., 2021); therefore, the large intensity increase of 9 cm s^−1^ per stage (i.e., 5.4 m min^−1^) adopted in the incremental test is not surprising. Although the BALB/c (Nu/Nu) mice were adapted to the treadmill exercise, we opted for 2.0 m min^−1^ increments to avoid premature exhaustion within the new stage, allowing a precise determination of the V_Peak_.

The reproducibility indexes were high for most of the samples. The consistency of the exhaustion criterion during the incremental test was critical to achieving this positive result. The same researcher conducted the test and retest (and also the retest after the intervention) protocol within the same BALB/c (Nu/Nu) mice, avoiding personal variations regarding the test interruption. Future research must carefully consider this factor since it can impact the VPeak determination. The large reduction presented by animals 14 and 15 may be attributed to a non-complete recovery between test and retest. This hypothesis, however, is not supported by the literature, given the information absence on the recovery capacity of BALB/c (Nu/Nu) mice after physical effort. Future methodological studies can shed light on this issue by comparing the test and retest parameters within 48 or 72-h of recovery between efforts. However, our results support the conclusion that 48-h was sufficient to assure high reproducibility results for 86% of the sample.

### Experiment 2—Sensitivity of the incremental test parameters to the individualized training

Although experiments with rodents submitted to physical training allow a refined control of factorial variables, individualized prescription and load monitoring are recurrently overlooked. This perspective enables deep interpretations of the physical training on stress biomarkers (de Araujo et al., 2012) and molecular adaptations (Rodrigues et al., 2021; Forte et al., 2022). Previous studies have submitted BALB/c (Nu/Nu) mice to physical training lasting 8 (Jones et al., 2005; Hagar et al., 2019) or 12 weeks (Lee et al., 2017; Lee et al., 2018). Despite the important data provided by these reports on breast tumors, none have provided a deep analysis of the training parameters during the exercise intervention. This interpretation is relevant for any rodent, but special attention must be given to BALB/c (Nu/Nu) mice. It is widely accepted that prolonged and intense physical exercise may cause an open window of immunosuppression during recovery (Peake et al., 2017). Given the immunodeficient feature of BALB/c (Nu/Nu) mice, the intensity prescription must be carefully considered to enable a refined interpretation of what is being considered “intense” for these animals. Otherwise, the immunosuppression window can increase, leading to infections and premature deaths of these animals.

Previous studies have carried out an exercise with progressive but not individualized intensity (Jones et al., 2005; Lee et al., 2017; Lee et al., 2018; Hagar et al., 2019). After some acclimatization to the training, the 18 m min^−1^ was chosen in most of these reports, since it corresponds to 70–75% of murine VO_2max_ (Fernando et al., 1993). This range, however, was determined with C3H/H, Swiss Webster, and B6D2 strains of female mice, and not with BALB/c (Nu/Nu) mice. Considering all mice evaluated in this study, this intensity has over or underestimated the V_Peak_ for 19% (V_Peak_ = 15.4 ± 1.4 m min^−1^) and 56% (V_Peak_ = 22.1 ± 2.9 m min^−1^), respectively; only five animals presented the V_Peak_ near 18 m min^−1^. This reinforces the necessity of conducted and controlled and individualized training for BALB/c (Nu/Nu) mice.

Our study is the pioneer in this context. The 70% of the VPeak study was chosen based on the positive improvements observed in Swiss mice after 4 weeks of training (Picoli et al., 2018). In 85% of the days (17 out of 20), the BALB/c (Nu/Nu) mice from the EG completed at least 90% of the predicted load, indicating that the intensity at 70% of the V_Peak_ is a valid maker for training prescription. Because the training load was not changed—considering that was not the study’s major purpose—the animals may have acclimated to the exercise load, resulting in an increase in monotony and a decrease in strain in the last week. Therefore, future experiments with longer exercise interventions should perform a retest after 1 month to adjust the exercise intensity.

Animals ingested more food and water on the weekends. Jeong et al. (2013) found that constraint stress affects body weight and food intake by altering canonical food intake-related genes. Despite the factors associated with the modulations of food and water are beyond the main goal of this study, it is possible to suggest that higher food and water intake is associated with less stress generated by the decreased circulation of researchers. The higher food and water intake of the EG in some weeks was not surprising given the previous hypothesis associating physical activity and energy expenditure (Hayes and Garland, 1995; Koteja, 2000), which was likely affected by the physical training. These factors, on the other hand, did not affect the animal’s body weight.

The V_Peak_ is highly associated with endurance performance and requires large participation of the aerobic metabolism (Bosquet et al., 2002). In humans, this parameter can be used to predict performance over different distances (Morgan et al., 1989; Stratton et al., 2009). BALB/c (Nu/Nu) mice’s physical performance was improved after the training, confirming the VPeak as a robust parameter to detect improvements in the aerobic metabolism. Apart from the confirmation of our second hypothesis, it is important to state that the animals in the CG had a significant decrement in the V_Peak_ and total time. Such results agree with recent studies showing the impact of housing conditions on the physiological and behavioral features of mice (Scariot et al., 2019; Scariot et al., 2021; Scariot et al., 2022). BALB/c (Nu/Nu) mouse was housed in standard but also teemed as “small” cages. The mentioned studies conclude that this limited space may reduce spontaneous physical activity, mitigating locomotion and its beneficial improvements in aerobic capacity. This can explain the reduced V_Peak_ in the CG, but most important, trigger an alert for future studies involving the physical training of Balb/c (Nu/Nu).

### Limitations and future perspectives

The results of this report must be interpreted in light of its limitations. Based on the immunodeficient feature of BALB/c (Nu/Nu) mice, we opted to not perform invasive measurements. This limits our discussion in terms of physiological and molecular adaptations from physical exercise in these animals. Furthermore, our conclusions are limited to the effects of training at 70% of the VPeak. Our design cannot assure that other intensities provide the same outcome. Moreover, we did not conduct a follow-up analysis to verify the effects of the detraining period. Future studies can shed light on these issues by comparing the different intensities effects on BALB/c (Nu/Nu) mice VPeak. Follow-up studies evaluating mortality rate and the aerobic capacity after the training are welcome to transpose this design to humans diagnosed with cancer and limited to physical exercise due to the therapies. Further, the housing effects on physiological and behavioral features of BALB/c (Nu/Nu) mice can provide valuable data for future experiments with these mice. Lastly, both groups presented homoscedasticity even with male and female animals. Based on this result, sex comparisons were not performed for two main reasons. The number of animals in each group will be largely decreased, reducing the statistical outcomes. Moreover, since the variance between groups was similar, sex is likely to not impact the main results obtained in this study. However, future studies with a large number of animals can verify if sex is a factor that impacts the V_Peak_ reproducibility and sensitivity to training effects.

## Conclusion

We have demonstrated that V_Peak_ of BALB/c (Nu/Nu) mice can be easily obtained by a non-invasive incremental test. For 86% of our sample, this parameter was reproducible with a low bias. Moreover, the V_Peak_ was valid to individualize the exercise intensity and detect improvements in the aerobic metabolism after 1 month of physical training. Overall, our results support the use of this parameter in future experiments involving BALB/c (Nu/Nu) mice and exercise, refining the comprehension of manipulating the training variables in the contexts that this animal model is applied.

## Data Availability

The raw data supporting the conclusions of this article will be made available by the authors, without undue reservation.

## References

[B1] Aguiar A. S. Jr. Boemer G. Rial D. Cordova F. M. Mancini G. Walz R. (2010). High-intensity physical exercise disrupts implicit memory in mice: Involvement of the striatal glutathione antioxidant system and intracellular signaling. Neuroscience 171 (4), 1216–1227. 10.1016/j.neuroscience.2010.09.053 20888397

[B2] Bassett D. R. Jr. Howley E. T. (2000). Limiting factors for maximum oxygen uptake and determinants of endurance performance. Med. Sci. Sports Exerc. 32 (1), 70–84. 10.1097/00005768-200001000-00012 10647532

[B3] Billat V. L. Mouisel E. Roblot N. Melki J. (2005). Inter- and intrastrain variation in mouse critical running speed. J. Appl. Physiol. 98 (4), 1258–1263. 10.1152/japplphysiol.00991.2004 15542571

[B4] Bland J. M. Altman D. G. (1986). Statistical methods for assessing agreement between two methods of clinical measurement. Lancet 1 (8476), 307–310. 10.1016/s0140-6736(86)90837-8 2868172

[B5] Bosquet L. Leger L. Legros P. (2002). Methods to determine aerobic endurance. Sports Med. 32 (11), 675–700. 10.2165/00007256-200232110-00002 12196030

[B6] Budzynski W. Radzikowski C. (1994). Cytotoxic cells in immunodeficient athymic mice. Immunopharmacol. Immunotoxicol. 16 (3), 319–346. 10.3109/08923979409007097 7528237

[B7] Cohen J. (1994). The earth is round (p<. 05). Am. Psychol. 49, 997–1003. 10.1037/0003-066x.49.12.997

[B8] da Silva D. C. Orfali G. D. C. Santana M. G. Palma J. K. Y. Assuncao I. R. O. Marchesi I. M. (2022). Antitumor effect of isoquercetin on tissue vasohibin expression and colon cancer vasculature. Oncotarget 13, 307–318. 10.18632/oncotarget.28181 35145607 PMC8823695

[B9] de Araujo G. G. Papoti M. Dos Reis I. G. de Mello M. A. Gobatto C. A. (2012). Physiological responses during linear periodized training in rats. Eur. J. Appl. Physiol. 112 (3), 839–852. 10.1007/s00421-011-2020-2 21681481

[B10] de Oliveira C. T. P. Colenci R. Pacheco C. C. Mariano P. M. do Prado P. R. Mamprin G. P. R. (2019). Hydrolyzed rutin decreases worsening of anaplasia in glioblastoma relapse. CNS Neurol. Disord. Drug Targets 18 (5), 405–412. 10.2174/1871527318666190314103104 30868970

[B11] De Sousa R. A. L. Rodrigues C. M. Mendes B. F. Improta-Caria A. C. Peixoto M. F. D. Cassilhas R. C. (2021). Physical exercise protocols in animal models of alzheimer's disease: A systematic review. Metab. Brain Dis. 36 (1), 85–95. 10.1007/s11011-020-00633-z 33095371

[B12] De Souza C. T. Frederico M. J. da Luz G. Cintra D. E. Ropelle E. R. Pauli J. R. (2010). Acute exercise reduces hepatic glucose production through inhibition of the Foxo1/HNF-4alpha pathway in insulin resistant mice. J. Physiol. 588 (12), 2239–2253. 10.1113/jphysiol.2009.183996 20421289 PMC2911223

[B13] Diehl K. H. Hull R. Morton D. Pfister R. Rabemampianina Y. Smith D. (2001). European Centre for the Validation of Alternative, MA good practice guide to the administration of substances and removal of blood, including routes and volumes. J. Appl. Toxicol. 21 (1), 15–23. 10.1002/jat.727 11180276

[B14] Esteves M. Monteiro M. P. Duarte J. A. (2021). Role of regular physical exercise in tumor vasculature: Favorable modulator of tumor milieu. Int. J. Sports Med. 42 (5), 389–406. 10.1055/a-1308-3476 33307553

[B15] Ferioli M. Zauli G. Martelli A. M. Vitale M. McCubrey J. A. Ultimo S. (2018). Impact of physical exercise in cancer survivors during and after antineoplastic treatments. Oncotarget 9 (17), 14005–14034. 10.18632/oncotarget.24456 29568412 PMC5862633

[B16] Fernando P. Bonen A. Hoffman-Goetz L. (1993). Predicting submaximal oxygen consumption during treadmill running in mice. Can. J. Physiol. Pharmacol. 71 (10-11), 854–857. 10.1139/y93-128 8143245

[B17] Flanagan S. P. (1966). Nude', a new hairless gene with pleiotropic effects in the mouse. Genet. Res. 8 (3), 295–309. 10.1017/s0016672300010168 5980117

[B18] Forte L. D. M. de Almeida Rodrigues N. Cordeiro A. V. de Fante T. de Paula Simino L. A. de Souza Torsoni A. (2022). Effect of acute swimming exercise at different intensities but equal total load over metabolic and molecular responses in swimming rats. J. Muscle Res. Cell Motil. 43 (1), 35–44. 10.1007/s10974-022-09614-4 35084659

[B19] Giavazzi R. Jessup J. M. Campbell D. E. Walker S. M. Fidler I. J. (1986). Experimental nude mouse model of human colorectal cancer liver metastases. J. Natl. Cancer Inst. 77 (6), 1303–1308. 3467119

[B20] Gobatto C. A. Manchado-Gobatto F. B. Carneiro L. G. de Araujo G. G. Reis I. G. M. (2009). Maximal lactate steady state for aerobic evaluation of swimming mice. Comp. Exerc. Physiol. 6 (3), 99–103. 10.1017/s1755254009990109

[B21] Hagar A. Wang Z. Koyama S. Serrano J. A. Melo L. Vargas S. (2019). Endurance training slows breast tumor growth in mice by suppressing Treg cells recruitment to tumors. BMC Cancer 19 (1), 536. 10.1186/s12885-019-5745-7 31164094 PMC6549262

[B22] Hayes J. P. Garland T. Jr. (1995). The evolution of endothermy: Testing the aerobic capacity model. Evolution 49 (5), 836–847. 10.1111/j.1558-5646.1995.tb02320.x 28564873

[B23] Hojman P. Gehl J. Christensen J. F. Pedersen B. K. (2018). Molecular mechanisms linking exercise to cancer prevention and treatment. Cell Metab. 27 (1), 10–21. 10.1016/j.cmet.2017.09.015 29056514

[B24] Idorn M. Thor Straten P. (2017). Exercise and cancer: From "healthy" to "therapeutic. Cancer Immunol. Immunother. 66 (5), 667–671. 10.1007/s00262-017-1985-z 28324125 PMC5406418

[B25] Issacson J. M. Cattanach B. M. (1962). Report mouse. News Lett. 192 (27), 31–32.

[B26] Jeong J. Y. Lee D. H. Kang S. S. (2013). Effects of chronic restraint stress on body weight, food intake, and hypothalamic gene expressions in mice. Endocrinol. Metab. 28 (4), 288–296. 10.3803/EnM.2013.28.4.288 PMC3871039 24396694

[B27] Jones L. W. Eves N. D. Courneya K. S. Chiu B. K. Baracos V. E. Hanson J. (2005). Effects of exercise training on antitumor efficacy of doxorubicin in MDA-MB-231 breast cancer xenografts. Clin. Cancer Res. 11 (18), 6695–6698. 10.1158/1078-0432.CCR-05-0844 16166449

[B28] Juul P. Christensen H. B. Hougen H. P. Svendsen O. Thygesen P. Rygaard J. (1992). Athymic experimental animals in pharmaco-immunological research. Toxicol. Lett. 64-65, 85–92. 10.1016/0378-4274(92)90176-k 1471239

[B29] Kelland L. R. (2004). Of mice and men: Values and liabilities of the athymic nude mouse model in anticancer drug development. Eur. J. Cancer 40 (6), 827–836. 10.1016/j.ejca.2003.11.028 15120038

[B30] Koteja P. (2000). Energy assimilation, parental care and the evolution of endothermy. Proc. Biol. Sci. 267 (1442), 479–484. 10.1098/rspb.2000.1025 10737405 PMC1690555

[B31] Kraemer M. B. Priolli D. G. Reis I. G. M. Pelosi A. C. Garbuio A. L. P. Messias L. H. D. (2022). Home-based, supervised, and mixed exercise intervention on functional capacity and quality of life of colorectal cancer patients: A meta-analysis. Sci. Rep. 12 (1), 2471. 10.1038/s41598-022-06165-z 35169171 PMC8847564

[B32] Kregel K. C. Allen D. L. Booth F. W. Fleshner M. R. Henriksen E. J. Musch T. I. (2006). in Resource book for the design of animal exercise protocols. Editor Society A. P..

[B33] Kuipers H. Rietjens G. Verstappen F. Schoenmakers H. Hofman G. (2003). Effects of stage duration in incremental running tests on physiological variables. Int. J. Sports Med. 24 (7), 486–491. 10.1055/s-2003-42020 12968205

[B34] Lee B. S. Choi E. J. So W. Y. (2018). Cytochrome expression in breast cancer xenograft mice after 12 Weeks of treadmill exercise. Iran. J. Public Health 47 (5), 759–761. 29922622 PMC6005977

[B35] Lee B. S. So W. Y. Chung W. Choi E. J. (2017). Suppression of expression levels of constitutive androstane receptor by moderate exercise in BALB/c nude mice with breast cancer. Iran. J. Public Health 46 (8), 1154–1155. 28894724 PMC5575402

[B36] Liu X. Liu J. Guan Y. Li H. Huang L. Tang H. (2012). Establishment of an orthotopic lung cancer model in nude mice and its evaluation by spiral CT. J. Thorac. Dis. 4 (2), 141–145. 10.3978/j.issn.2072-1439.2012.03.04 22833819 PMC3378238

[B37] Lonbro S. Wiggins J. M. Wittenborn T. Elming P. B. Rice L. Pampo C. (2019). Reliability of blood lactate as a measure of exercise intensity in different strains of mice during forced treadmill running. PLoS One 14 (5), e0215584. 10.1371/journal.pone.0215584 31050686 PMC6499470

[B38] Ludtke D. D. Siteneski A. Galassi T. O. Buffon A. C. Cidral-Filho F. J. Reed W. R. (2020). High-intensity swimming exercise reduces inflammatory pain in mice by activation of the endocannabinoid system. Scand. J. Med. Sci. Sports 30 (8), 1369–1378. 10.1111/sms.13705 32358841

[B39] Manchado-Gobatto F. B. Contarteze R. V. L. Papoti M. de Araujo G. G. Mello M. A. R. (2010). Determination of critical velocity and anaerobic capacity of running rats. J. Exerc. Physiology Online 13, 40–49.

[B40] Messias L. H. D. Gobatto C. A. Beck W. R. Manchado-Gobatto F. B. (2017). The lactate minimum test: Concept, methodological aspects and insights for future investigations in human and animal models. Front. Physiol. 8, 389. 10.3389/fphys.2017.00389 28642717 PMC5463055

[B41] Mitruka B. M. Rawnsley H. M. (1977). Clinical biochemical and hematological reference values in normal experimental animals. USA: Masson Publishing.

[B42] Morgan D. W. Baldini F. D. Martin P. E. Kohrt W. M. (1989). Ten kilometer performance and predicted velocity at VO2max among well-trained male runners. Med. Sci. Sports Exerc. 21 (1), 78–83. 10.1249/00005768-198902000-00014 2927305

[B43] Moss J. L. Pinto C. N. Srinivasan S. Cronin K. A. Croyle R. T. (2020). Persistent poverty and cancer mortality rates: An analysis of county-level poverty designations. Cancer Epidemiol. Biomarkers Prev. 29 (10), 1949–1954. 10.1158/1055-9965.EPI-20-0007 32998949 PMC7534551

[B44] Osborne C. K. Hobbs K. Clark G. M. (1985). Effect of estrogens and antiestrogens on growth of human breast cancer cells in athymic nude mice. Cancer Res. 45 (2), 584–590. 3967234

[B45] Pantelouris E. M. (1968). Absence of thymus in a mouse mutant. Nature 217 (5126), 370–371. 10.1038/217370a0 5639157

[B46] Peake J. M. Neubauer O. Walsh N. P. Simpson R. J. (2017). Recovery of the immune system after exercise. J. Appl. Physiol. 122 (5), 1077–1087. 10.1152/japplphysiol.00622.2016 27909225

[B47] Picoli C. C. Gilio G. R. Henriques F. Leal L. G. Besson J. C. Lopes M. A. (2020). Resistance exercise training induces subcutaneous and visceral adipose tissue browning in Swiss mice. J. Appl. Physiol. 129 (1), 66–74. 10.1152/japplphysiol.00742.2019 32501777

[B48] Picoli C. C. Romero P. Gilio G. R. Guariglia D. A. Tofolo L. P. de Moraes S. M. F. (2018). Peak velocity as an alternative method for training prescription in mice. Front. Physiol. 9, 42. 10.3389/fphys.2018.00042 29467664 PMC5808179

[B49] Rodrigues N. A. Gobatto C. A. Forte L. D. M. Sousa F. A. B. Torsoni A. S. Fante T. (2021). Load-matched acute and chronic exercise induce changes in mitochondrial biogenesis and metabolic markers. Appl. Physiol. Nutr. Metab. 46 (10), 1196–1206. 10.1139/apnm-2020-1053 33779293

[B50] Rodrigues N. A. Torsoni A. S. Fante T. Dos Reis I. G. Gobatto C. A. Manchado-Gobatto F. B. (2017). Lactate minimum underestimates the maximal lactate steady-state in swimming mice. Appl. Physiol. Nutr. Metab. 42 (1), 46–52. 10.1139/apnm-2016-0198 28006434

[B51] Scariot P. P. Gobatto C. A. Polisel E. E. Gomes A. E. Beck W. R. Manchado-Gobatto F. B. (2022). Early-life mice housed in standard stocking density reduce the spontaneous physical activity and increase visceral fat deposition before reaching adulthood. Lab. Anim. 236772211065915, 002367722110659. 10.1177/00236772211065915 35062839

[B52] Scariot P. P. M. Manchado-Gobatto F. B. Prolla T. A. Masselli Dos Reis I. G. Gobatto C. A. (2019). Housing conditions modulate spontaneous physical activity, feeding behavior, aerobic running capacity and adiposity in C57BL/6J mice. Horm. Behav. 115, 104556. 10.1016/j.yhbeh.2019.07.004 31310763

[B53] Scariot P. P. M. Manchado-Gobatto F. B. Van Ginkel P. R. Prolla T. A. Gobatto C. A. (2021). Aerobic training associated with an active lifestyle exerts a protective effect against oxidative damage in hypothalamus and liver: The involvement of energy metabolism. Brain Res. Bull. 175, 116–129. 10.1016/j.brainresbull.2021.07.018 34303768

[B54] Statista (2022). Global oncology spending from 2011 to 2022. Retrieved from https://www.statista.com/statistics/696208/oncology-costs-worldwide/.

[B55] Stratton E. O'Brien B. J. Harvey J. Blitvich J. McNicol A. J. Janissen D. (2009). Treadmill velocity best predicts 5000-m run performance. Int. J. Sports Med. 30 (1), 40–45. 10.1055/s-2008-1038761 19202577

[B56] Szadvari I. Krizanova O. Babula P. (2016). Athymic nude mice as an experimental model for cancer treatment. Physiol. Res. 65 (4), S441–S453. 10.33549/physiolres.933526 28006926

[B57] van Weerden W. M. Romijn J. C. (2000). Use of nude mouse xenograft models in prostate cancer research. Prostate 43 (4), 263–271. 10.1002/1097-0045(20000601)43:4<263::aid-pros5>3.0.co;2-i 10861745

[B58] Volpe R. Kasuga Y. Akasu F. Morita T. Yoshikawa N. Resetkova E. (1993). The use of the severe combined immunodeficient mouse and the athymic "nude" mouse as models for the study of human autoimmune thyroid disease. Clin. Immunol. Immunopathol. 67 (2), 93–99. 10.1006/clin.1993.1050 8519094

[B59] Wanner S. P. Costa K. A. Soares A. D. Cardoso V. N. Coimbra C. C. (2014). Physical exercise-induced changes in the core body temperature of mice depend more on ambient temperature than on exercise protocol or intensity. Int. J. Biometeorol. 58 (6), 1077–1085. 10.1007/s00484-013-0699-y 23857354

[B60] Yoysungnoen P. Wirachwong P. Changtam C. Suksamrarn A. Patumraj S. (2008). Anti-cancer and anti-angiogenic effects of curcumin and tetrahydrocurcumin on implanted hepatocellular carcinoma in nude mice. World J. Gastroenterol. 14 (13), 2003–2009. 10.3748/wjg.14.2003 18395899 PMC2701520

[B61] Zhao M. Tang S. N. Marsh J. L. Shankar S. Srivastava R. K. (2013). Ellagic acid inhibits human pancreatic cancer growth in Balb c nude mice. Cancer Lett. 337 (2), 210–217. 10.1016/j.canlet.2013.05.009 23684930

